# Mapping Antimalarial Drug Resistance in Mozambique: A Systematic Review of *Plasmodium falciparum* Genetic Markers Post-ACT Implementation

**DOI:** 10.3390/ijms252413645

**Published:** 2024-12-20

**Authors:** Celso Raul Silambo Chaves, Clemente da Silva, Acácio Salamandane, Fatima Nogueira

**Affiliations:** 1 Global Health and Tropical Medicine (GHTM), Associate Laboratory in Translation and Innovation Towards Global Health (LA-REAL), Instituto de Higiene e Medicina Tropical (IHMT), Universidade NOVA de Lisboa (UNL), Rua da Junqueira 100, 1349-008 Lisboa, Portugal; a21002189@ihmt.unl.pt (C.R.S.C.); a21000955@ihmt.unl.pt (C.d.S.); 2 Faculdade de Ciências de Saúde, Universidade Lúrio, Campus Universitário de Marrere, Nampula 4250, Mozambique; salamandane@gmail.com

**Keywords:** *Plasmodium falciparum*, malaria drug resistance, molecular markers, artemisinin-based combination therapy, Mozambique

## Abstract

Malaria continues to be a significant public health burden in many tropical and subtropical regions. Mozambique ranks among the top countries affected by malaria, where it is a leading cause of morbidity and mortality, accounting for 29% of all hospital deaths in the general population and 42% of deaths amongst children under five. This review presents a comparative analysis of data on five critical genes associated with antimalarial drug resistance: *pfmdr1*, *pfcrt*, *pfk13*, *pfdhfr*, and *pfdhps*, along with the copy number variation (CNV) in genes *pfmdr1* and *pfpm2/3*. These are genes associated with parasite response to antimalarials currently used to treat uncomplicated *P. falciparum* malaria in Mozambique. The review synthesizes data collected from published studies conducted in Mozambique after the introduction of artemisinin-based combination therapies (ACTs) (2006) up to June 2024, highlighting the presence or absence of mutations in these genes across Mozambique. We aimed at mapping the prevalence and distribution of these molecular markers across the country in order to contribute to the development of targeted interventions to sustain the efficacy of malaria treatments in Mozambique. Four databases were used to access the articles: PubMed, Science Direct, Scopus, and Google scholar. The search strategy identified 132 studies addressing malaria and antimalarial resistance. Of these, 112 were excluded for various reasons, leaving 20 studies to be included in this review. Children and pregnant women represent the majority of target groups in studies on all types of antimalarials. Most studies (87.5%) were conducted in the provinces of Maputo and Gaza. The primary alleles reported were *pfcrt* CVMNK, and in the most recent data, its wild-type form was found in the majority of patients. A low prevalence of mutations in the *pfk13* gene was identified reflecting the effectiveness of ACTs. In *pfk13*, only mutation A578S was reported in Niassa and Tete. CNVs were observed in studies carried out in the south of Mozambique, with a frequency of 1.1–5.1% for *pfmdr1* and a frequency of 1.1–3.4% for *pfpm2*. This review indicates that molecular markers linked to malaria resistance show considerable variation across provinces in Mozambique, with most up-to-date data accessible for Maputo and Gaza. In contrast, provinces such as Zambezia and Inhambane have limited data on several genes, while Nampula lacks data on all drug resistance markers.

## 1. Introduction

Malaria remains one of the most pressing public health challenges in many tropical and subtropical countries, impacting millions of lives across the region [1,2,3]. Mozambique faces a substantial malaria challenge, being one of the countries with the highest number of cases and ranking fourth globally in terms of the malaria burden [4]. To combat malaria effectively, Mozambique must address both the vector and the administration of antimalarial drugs [5].

The ongoing fight against malaria has been complicated by the emergence and spread of *Plasmodium falciparum* resistance to antimalarial drugs, which compromises the efficacy of treatment regimens and poses a significant threat to malaria control efforts [6,7,8]. Understanding the distribution of molecular markers of antimalarial resistance is essential for monitoring and managing drug resistance, revising treatment guidelines, and informing the development of new antimalarial drugs.

In Mozambique, as in other parts of sub-Saharan Africa, *P. falciparum* is the predominant malaria parasite. The region has seen various waves of drug resistance, particularly to chloroquine (CQ), sulfadoxine-pyrimethamine (SP), and, more recently, to artemisinin-based combination therapies (ACTs) [1]. Molecular markers have been instrumental in tracking and understanding these resistance patterns [1,9].

Chloroquine was the first-line treatment for uncomplicated malaria in Mozambique for almost 50 years until 2004, when sulfadoxine-pyrimethamine (SP) and amodiaquine (AQ) were introduced due the emergence and spread of resistance to chloroquine [10]. The primary molecular marker associated with chloroquine resistance is the *P. falciparum* drug resistance transporter (*pfcrt*) gene, particularly the single-nucleotide polymorphism (SNP) K76T [11]. Several studies have reported high frequencies of this mutation in Mozambique and neighboring countries such as Tanzania and Malawi, reflecting the extensive spread of CQ resistance (CQ-R) in the region [12,13,14]. The haplotype defined by specific mutations at amino acid positions 72–76 of *pfcrt*, CVIET, has been associated with CQ-R (in Africa), while the haplotype CVMNK is associated with CQ susceptibility (CQ-S) [15,16]. Following the decline in CQ efficacy, artesunate plus SP was introduced in Maputo Province between 2004 and 2006 as the mainstay for malaria treatment [10]. However, during this pilot study, molecular markers associated with SP resistance (SNPs in the genes dihydropteroate synthase, *pfdhps*, and dihydrofolate reductase, *pfdhfr*) increased dramatically [10,17,18]. SP resistance is associated with the SNPs A16V, N51I, C59R, S108N, and I164L in the *pfdhfr* gene, which confer resistance to pyrimethamine, and I431V, S436A/F, A437G, K540E, A581G, and A613S/T in the *pfdhps*, which confer resistance to sulfadoxine [17,19]. Parasites with multiple SNPs in both *pfdhfr* and *pfdhps* were categorized as follows: a quadruple mutant (*pfdhfr* 51I + 59R + 108N and *pfdhps* 437G [IRNG]) was classified as “partially resistant”; a quintuple mutant (*pfdhfr* 51I + 59R + 108N and *pfdhps* 437G + 540E [IRNGE]) as “fully resistant”; and a sextuple mutant (*pfdhfr* 51I + 59R + 108N and *pfdhps* 437G + 540E + 581G or 613S/T [IRNGEG or IRNGES/T]) as “super resistant” [20].

These findings led to a change in the national malaria treatment policy in 2008 to the use of ACTs. In Mozambique, the recommended treatment for uncomplicated *P. falciparum* malaria consists of: artemether-lumefantrine (AMT-LUM) (first line of treatment since 2006 [21], artesunate-sulfadoxine and pyrimethamine (AS-SP), artesunate-amodiaquine (AS-AQ), artesunate-mefloquine (AS-MEF), dihydroartemisinin-piperaquine (DHA-PPQ), and artesunate-pyronaridine AS-PY [10,22]. In ACTs, artemisinin derivatives (short half-life; <6 h) are combined with long-acting antimalarial drugs like AQ, MEF, PPQ, LUM, and pyronaridine (PY) to treat uncomplicated malaria [22,23,24]. Regarding ACT partner drugs, the primary genes associated with resistance are *pfcrt*, *pfmdr1*, *pfpm2/3*, and the above-mentioned *pfdhfr* and *pfdhps* [25]. Multiple copies (or copy number variations, CNV) of the *P. falciparum* multidrug resistance 1—*pfmdr1* gene are established markers for resistance to MEF (MEF-R) [26,27]. Additionally, SNPs in *pfmdr1* have been linked to altered parasite tolerance or susceptibility to several antimalarial drugs, including quinine (QN), AQ, CQ, MEF, and lumefantrine (LUM) [28]. The key *pfmdr1* SNPs associated with drug resistance include N86Y, Y184F, S1034C, and N1024D [29,30,31,32,33,34]. The N86Y mutation is related to increased CQ-R and increased sensitivity to MEF [35]. Parasites carrying *pfmdr1* haplotype 86Y Y184 show increased susceptibility to LUM and MEF [36]. The role of the *pfmdr1* N86, 184F, and 1246D alleles, as well as *pfmdr1* CNV, in *P. falciparum’s* response to AMT-LUM remains debated [37].

In recent years, resistance to ACTs has been reported in Southeast Asia in 2008 [25,38,39]. Recently, SNPs associated with resistance to artemisinins in Africa [40,41,42] were identified. Resistance to artemisinin derivatives is characterized by delayed parasite clearance times and is linked to SNPs in the Kelch13 protein coded by the gene *pfk13*. In particular, F446I, N458Y, M476I, Y493H, R539T, I543T, P553L, R561H, and C580Y are currently considered validated molecular markers of drug resistance by WHO [38,39,41]. This study’s objective is to provide a comprehensive analysis of prevalence and distribution of the molecular markers of antimalarial resistance in Mozambique. By mapping the prevalence and distribution of these markers, this research aims to contribute to supporting the development of targeted interventions to maintain the effectiveness of malaria treatments in Mozambique.

## 2. Methods

### 2.1. Selection of Relevant Literature

This study was conducted according to the recommendations of the Preferred Reporting Items for Systematic Reviews (PRISMA) [43,44]. Briefly, the search terms and criteria for the inclusion or exclusion were previously defined to be searched across various databases. After conducting the article search, the selection of the studies based on the inclusion criteria were assessed by two independent researchers. In cases of disagreement, a third researcher was consulted to solve the dispute. Following the selection of articles for inclusion in the study, a thorough analysis was conducted to extract the most important findings and conclusions. Subsequently, these data were organized and presented in tables or figures. The databases searched were Scopus, PubMed, and Web of Science, in addition to isolated searches for relevant articles found on Google Scholar. A total of 132 articles published from 2007 to July 2024 complied with the inclusion criteria in the title, keywords, or summary. Aligning with the national rollout of ACTs in 2006 [21], and to capture the progress made in molecular monitoring of antimalarial resistance, a 17-year study period was chosen.

### 2.2. Eligibility Criteria of Studies Include in the Review

The inclusion criteria were all original articles addressing molecular marker of antimalarial drug resistance published in indexed journals (PubMed, Science Direct, Scopus, and Google Scholar) using the keywords: “*pfpm2/3* OR *pfmdr1* OR *pfk13* OR *pfdhps* OR *pfdhfr* OR ‘*pfcrt*’ OR ‘copy number variation’, AND ‘Mozambique”.

### 2.3. Screening and Data Extraction

The articles selected for the study were exported to Microsoft Excel to remove duplicates. The selection of articles was carried out by reading the titles and abstracts and then the full text. The studies were systematized by authorship, year, sociodemographic data, sample size, allele or gene, amino acid, haplotype, type of mutation, CNV, respective prevalence, antimalarial drug, and main conclusions. The quality assessments of the studies were performed using a tool for assessing risk of bias in randomized studies (Cochrane ROB2) and a tool for assessing risk of bias in non-randomized studies (ROBINS-I).

## 3. Results and Discussion

### 3.1. Basic Characteristics of Included Studies

**Figure 1:**
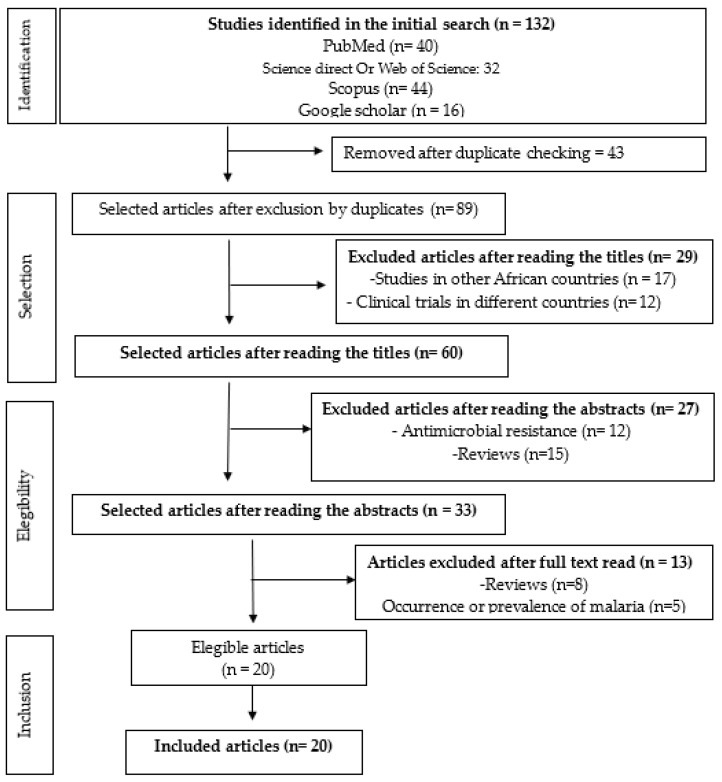
PRISMA diagram of the systematic review. Steps followed by this systematic review according to the PRISMA (“Preferred Reporting Items for Systematic Reviews and Meta-Analyses”) guidelines.

The search strategy identified 132 studies, from which 43 duplicates were removed. After screening titles and abstracts, 56 studies were excluded. Of the remaining 33 studies, 13 were excluded after full-text review, leaving 20 studies for inclusion in this review (Figure 1).

Children under 5 years of age and pregnant women comprise most targeted groups for all types of antimalarials, followed by children and adolescents up to 15 years. Few studies have focused on adult patients. The genes *pfdhfr* and *pfdhps*, associated with SP resistance, were identified in studies focusing on patients of all ages and sexes [45,46]. Regarding the gene *pfk13*, associated with resistance to artemisinin derivatives, the study by Da Silva [47] included both children and adults of both sexes, while the study by Escobar [48] was focused on adult patients of both sexes.

**Figure 2:**
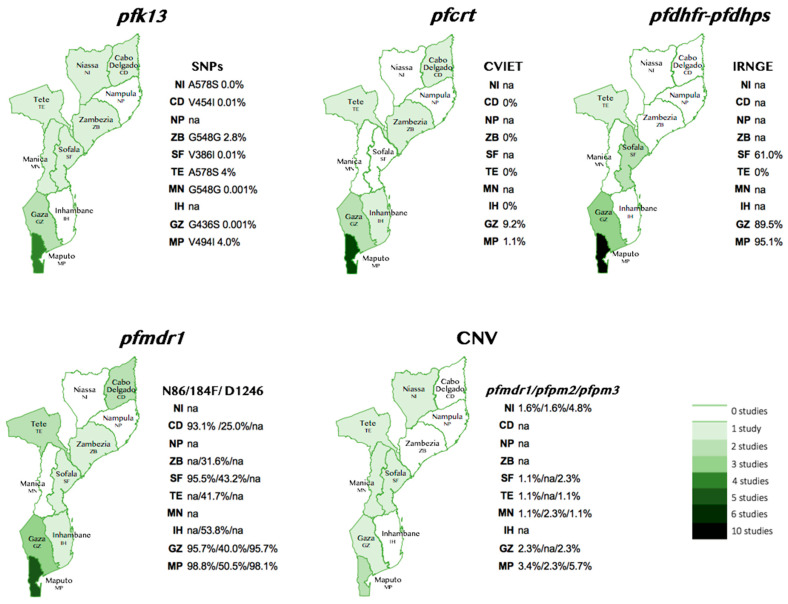
Geographical distribution of studies and the most recent data available for each molecular marker. Colored rectangles represent the number of studies identified for each molecular marker; CNV, copy number variation; SNPs, single nucleotide polymorphisms; CVIET, amino acid positions 72-76 of *pfcrt*; IRNGE, amino acid positions 51I + 59R + 108N of *pfdhfr* and *pfdhps* 437G + 540E of *pfdhps*; na, not available; NI, Niassa; CD, Cabo Delgado; NP, Nampula; ZB, Zambesia; SF, Sofala; TE, Tete; MN, Manica; IH, Inhambane; GZ, Gaza; MP, Maputo.

The information about the 20 studies included in this review is summarized in Table 1 and Figure 2 and detailed in the Supplementary Materials (Tables S1–S5). Most studies (17/20; 85%) were conducted in southern Mozambique, specifically in the provinces of Maputo and Gaza (Table 1). It is important to emphasize that 40% (8/20) of the total articles included in this study addressed three genes (*pfcrt*, *pfdhfr*, and *pfdhps*, *pfmdr1*, *pfk13* and CNVs *pfpm2/pfpm3/pfmdr1*) in different provinces [49,50,51,52,53,54,55] (detailed in Supplementary Materials). 

A total of nine studies (45%) monitored the *pfcrt* gene (associated with CQ-R) (Table 1): six in Maputo; two in Gaza; and one for Tete, Zambezia, Cabo Delgado, and Inhambane. *pfdhr* and *pfdhps* genes, associated with SP resistance, were found in 13 studies (65%); 10 of these studies were conducted in Maputo (Table 1). Of these 13 studies, 9 involved children, 4 pregnant women, and 1 adults (Table S2).

A total of nine studies were found addressing the *pfmdr1* gene (Table 1). Five of these focused on Maputo and two on Gaza, with five involving children and four pregnant women (Table S3). Five studies were found examining the *pfk13* gene (Table 1), including two involving adults and pregnant women and three involving children, mainly in Maputo (Table S4). Finally, three studies investigating CNV were identified; all included pregnant women and children, with two in Maputo and one in Niassa, Manica, Sofala, Tete, and Gaza, respectively. This overview highlights a concentration of studies in the Maputo and Gaza provinces (in the south of the country; Figure 2) and a predominance of research involving children and pregnant women (detailed in Supplementary Materials).

Malaria remains a significant public health concern in Mozambique, with *Plasmodium falciparum* being the predominant species responsible for the disease [65]. Understanding the genetic variants associated with drug resistance is crucial for developing effective treatment strategies and transmission control of the disease. This review reveals substantial regional variability in genetic mutations associated with malaria drug resistance in Mozambique (Figure 2). While the data are more robust for Maputo and Gaza, significant gaps remain for other provinces, underscoring the need for further research to monitor genetic variations over time. For instance, research on *pfcrt* gene polymorphisms, which encode CQ-R, primarily focused on two southern provinces. The prevalence of *pfcrt* gene was detected as between 40 and 84% of patients [10,56,61]. In the past, these data were important for changing the malaria treatment, which at the time was based on chloroquine, in line with what was happening in all malaria-endemic countries [66]. In Gaza and Maputo, a moderate to high prevalence of the 76T *pfcrt* SNP (CQ-R) was found during the first years after the introduction of ACTs [10,55]. However, more recent studies conducted in the same provinces after the discontinuation of chloroquine (samples collected 2017–2019) have identified a high prevalence of CQ-S *P. falciparum* genotypes [52,53,54]. This shift suggests a reduced selective pressure from CQ. Similar trends have been observed in other sub-Saharan African countries, including Kenya, Malawi, Sierra Leone, Ghana, Angola, and Ivory Coast, where CQ-S *P. falciparum* genotypes have re-emerged [67,68,69,70].

### 3.2. Antimalarial Resistance Associated Polymorphisms

#### 3.2.1. *pfcrt*

The majority of the studies (87.5%) addressing the *pfcrt* gene were conducted in the provinces of Maputo and Gaza (Table 1 and Table S1), with only one study addressing multiple provinces, namely Inhambane, Zambezia, Tete, and Cabo Delgado (see Table S1). The most recent evaluation of *pfcrt* CVIET haplotype was from 2024 and revealed prevalences of 1.1% in Maputo; 9.2% in Gaza; and 0% in Inhambane, Zambezia, Tete and Cabo Delgado (see Figure 2 and Table S1). The most prevalent haplotype was CVMNK (CR-susceptible), found in 92.2% of patients sampled in Cabo Delgado, Tete, Zambezia, and Inhambane in 2021 [53]. CVIET (CR-resistant) was reported in 7.8% of patients sampled in Inhambane, Zambezia, Tete, and Cabo Delgado [56], and 76T was found in 84% of adult patients of both genders in Maputo province in 2024 [50], 48.8% in children aged 2 to 3 months, and 46.4% in pregnant women [62]. The gene *pfcrt* confers resistance to a wide range of quinoline and quinoline-like antimalarial drugs in *P. falciparum*, with local drug histories driving its evolution and, thus, the drug transport specificities. For example, the change in prescription practice from CQ to PPQ in Southeast Asia has resulted in *pfcrt* variants that carry additional SNPs (H97Y, F145I, M343L, or G353V), leading to PPQ resistance [71]. There is a notable gap in the current understanding of specific *pfcrt* SNPs in Mozambique, particularly the ones associated to PPQ-R in Southeast Asia. Evaluating these markers in Mozambique could provide essential information for updating malaria treatment guidelines and managing potential PPQ-R as drug policies shift in the country.

#### 3.2.2. *pfdhr* and *pfdhps*

The prevalence of the SP-resistance haplotype IRNGE was high in Maputo (95.1%) and Gaza (89.5%) (Figure 2). The main studied mutations occurred at amino acid positions 51, 59, 108, 164, 437, 540, and 58, either individually or in combination within the *pfdhfr* or *pfdhps* genes, resulting in multidrug resistance haplotypes (see Table S2). Taking into account the most recent results, in the Maputo province, the most frequent haplotype was IRNGE, with 95.1% prevalence in samples collected in 2015–2019 [51] and 94.2% in samples collected in 2016–2019 (Table S2) [62]. In Gaza, the sextuple IRNGEG haplotype was observed in 8% of the samples and the quintuple IRNGE in 55% [72]. Studies from the central (Tete and Sofala) [53] and southern (Gaza and Maputo) [73] regions reported higher prevalence of SNPs in the *pfdhfr* or *pfdhps* in various combinations than the northern (Cabo Delgado) provinces [53] (Table S2).

In Mozambique, SP is used for intermittent preventive treatment in pregnancy (IPTp) and has been linked to the accumulation of SP-resistant mutations in *pfdhfr* and *pfdhps* [10,50,61]. This may facilitate the selection of resistant parasites due to the repeated exposure to SP. Nevertheless, despite widespread SP resistance, studies indicate that administering three or more doses of SP to pregnant women may still confer a protective benefit against *P. falciparum* [62]. The geographical distribution of *pfdhfr* and *pfdhps* SNPs studies in Mozambique reveals uneven coverage across provinces, with a significant focus on the southern region, particularly Maputo (10; Table S2), with a limited number of studies conducted in other provinces (three in Gaza [10,50,52], two in Sofala [53,60] and one in Cabo Delgado and Tete [53]; Table S2). The remaining five provinces do not have published information (Figure 2). In Maputo, high prevalence rates of mutations associated with SP-R were reported, such as 51I (36.6–88%) in *pfdhfr* and 59R (52.4–91%), 108N (50.4–99.2%), 540E (7.9–94.9), and 437G (42–96.2%) in *pfdhps*. The quintuple mutant IRNGE was reported in multiple studies with high prevalence, namely, 94.2% in samples from 2016 to 2019 [62] and 95.1% in samples from 2015 to 2019 [51] (Table S2). This underscores substantial SP resistance in Mozambique and follows the trend of other African countries, such as Ghana and Nigeria [74,75].

#### 3.2.3. *pfmdr1*

SNPs in *pfmdr1* were studied in all provinces except Niassa, Manica and Nampula (Figure 2 and Table S3). The latest reported prevalences of SNPs were N86, found in 93.1% in Cabo Delegado, 95.7% Gaza, 95.5% in Sofala, and 98.8% in Maputo, respectively, and 184F, reported in 41.7% in Tete, 43.2% in Sofala, 50.5% in Maputo, and 53.58% in Inhambane, respectively (Figure 2).

The *pfmdr1* encodes a protein involved in drug transport within the parasite and plays a key role in susceptibility to the key antimalarial ACTs. Although mutations in *pfmdr1* are not directly responsible for resistance to artemisinins, they influence the effectiveness of partner drugs, such as LUM or AQ, and the haplotype NFD has been associated with higher susceptibility to these partner drugs [29,30,31,32,33,34]. After Mozambique transitioned from chloroquine to ACTs for malaria control, the prevalence of *pfmdr1* mutations changed, with the NFD haplotype (amino acids 86/184/1246) variant becoming more common [52,54]. The current data reveal a significant geographical gap in the country regarding studies on the *pfmdr1* gene. Most research has been concentrated in Maputo (5) [51,52,55,57,63] and Gaza (3) [10,53,76]. There are limited data from other provinces, like Cabo Delgado and Tete (2) [53,54] or Zambezia, Inhambane (1) [5], and Sofala (1) [53]. This regional imbalance of studies leaves large parts of Mozambique underrepresented, especially in the northern and central provinces. For instance, no studies have been recorded in Nampula for *pfmdr1* (or any other molecular marker), and in Sofala, the only study available is based on samples collected nearly a decade ago (2015) [53]. The most recent studies have identified an appreciable prevalence of mutations in *pfmdr1*, namely, the SNPs N86 (98.8%) and 184F (75.4%) in Maputo (samples collected in 2015–2019) [51] and the haplotype NFD in Inhambane 74.4%, Cabo Delgado 66.7%, Tete 11.0%, or Zambezia 50.0% (samples collected in 2018) [54]. Similar trends have been observed in several other African countries [76,77,78,79].

#### 3.2.4. *pfk13*

Polymorphisms of *pfk13* associated with multidrug resistance in *P. falciparum* were investigated in five studies (Table S4). Most studies (75%) were conducted in eight provinces, except Inhambane and Nampula. Only one study examined multiple provinces (Figure 2 and Table S4). A low frequency of *pfk13* was observed in all provinces where studies were conducted (Figure 2). Maputo and Tete, with 4% each, were the provinces with the highest prevalence of *pfk13* SNPs (Figure 2). Notable, findings included the synonymous mutation at codon 469 (TGC to TGT) in one sample and at codon 548 (GGC to GGT) in three samples from Zambezia province (Mopeia city [54]). Two studies were identified for the SNPs: 494I [52] and 578S [52], both with 4% prevalence and both in samples from Maputo province [48] (Table S4). Neither of these two SNPs is currently considered a validated molecular marker of drug resistance by WHO [38,39,41].

The prevalence of *pfk13* SNPs varies by region; in 2019, it was 45.4% in Southeast Asia compared to a much lower prevalence of 7.6% in Africa [66]. In Mozambique, 8/10 provinces have evaluated the presence of *pfk13* SNPs, and none of the validated or candidate mutations have been identified so far. Similar findings have been reported in other African countries like Gabon [80], Senegal [81], Kenya, and Ethiopia [82], where low frequencies of *pfk13* SNPs have been observed. However, A578S was detected in samples from Niassa and Tete provinces [54,83], as well as in Uganda and Gabon [84]. The identification of independent emergence of *pfk13* SNPs (with partial resistance to ACTs) in the African region, especially in Rwanda and Uganda [85,86,87,88], highlights the importance of surveillance efforts to obtain genotypic data and map the extent of *pfk13* SNPs throughout the WHO African Region [89]. The recent detection of SNPs M476I, P553L, R561H, P574L, and C580Y in Africa serves as an early warning signal [40,41,42,90].

#### 3.2.5. Copy Number Variations in *pfmdr1* and *pfpm2/3*

Figure 2 summarizes the latest prevalence rates and primary study provinces, and Table S5 displays detailed data collected from various populations (children, adults, pregnant women) between 2015 and 2023. Only three studies were found investigating the prevalence of copy number variations (CNVs) in *pfmdr1* and *pfpm2* (Table 1). Two studies, Brown et al., 2024 [64] and Gupta et al., 2018 [53], covered multiple provinces, while the third study (Gupta et al., 2020 [52]) focused solely on Maputo province (Table S5). *Pfmdr1* CNV prevalence rates were as follows: 4.8% in the north (Niassa); between 1.1%, 2.3% in Tete, Manica, and Sofala (center); and 5.7% in the south (Maputo; Figure 2). Regarding plasmepsins (*pfpm2* and *pfpm3*) CNVs, prevalence rates were higher in the southern provinces of Gaza and Maputo (3.4% for *pfpm2* and 2.3% for *pfpm3*) compared to the northern and central provinces (Niassa, Tete, Manica, and Sofala), where the prevalence ranged from 1.1% to 1.6% for *pfpm2* and 1.6% to 2.3% for *pfpm3* (specifically 1.6% for *pfpm3* in Niassa and 2.3% in Manica).

There are only three studies assessing CNVs of *pfmdr1*, *pfpm2*, and *pfpm3* in Mozambique; one assessing all three [64]; and two assessing *pfmdr1* and *pfpm2* [52,53]. These revealed prevalence rates ranging from 1.1 to 5.7% for *pfmdr1*, 1.1 to 3.4% for *pfpm2*, and 1.6 to 2.3% for *pfpm3* [10,22,57]. Studies from Mozambique revealed a much lower prevalence of *pfmdr1* CNV than other African countries, namely Kenya (6.2%), Ghana (18%), Tanzania (10.2%), West Ethiopia (8.4%), and North of Ethiopia (54.14%) [76,91,92,93,94]. Observations from Mozambique, on the other hand, are in line with studies from other African countries like Nigeria or Democratic Republic of Congo, where increased CNV was not observed for *pfmdr1* [95,96]. Regarding *pfpm2* prevalence, the two studies recorded in Mozambique also reported much lower prevalences than others from Africa (7.7% in Tanzania [91] and 67.9% in Guinea Equatorial [97], but comparable to, e.g., Liberia or Uganda, increased copies of *pfpm2* were not observed [98,99]).

Copy number variation (CNV) has also been found to play a significant role in the development of antimalarial drug resistance. One copy of *pfmdr1* is associated with slower clearance of parasites after PPQ treatment as compared to more copies of *pfmdr1* [100], while having two copies of *pfpm2* is associated with slower clearance [101,102], after PPQ treatment. This inverse selection pressure argues in favor of keeping these molecular markers under constant surveillance.

## 4. Conclusions

Although malaria is endemic throughout the country, the central and northern regions of Mozambique have the highest incidences, especially the provinces of Zambezia, Nampula, and Cabo Delegado, which are most affected by the disease [103]. This situation may be associated with the fact that these are coastal provinces, with climatic conditions and socio-economic factors favorable for the proliferation of the malaria vector. However, most studies on monitoring molecular markers of resistance to antimalarials are concentrated in the southern region of the country.

Maputo province has had the highest number of and more up-to-date studies conducted (17), followed by Gaza (4) and Tete, Sofala, and Cabo Delgado (3). Other provinces such as Manica and Niassa (2), Zambezia, and Inhambane (1) have limited studies, while no studies have been reported from Nampula. This review highlights the concentration of research efforts primarily in Maputo, reflecting a potential need for further investigation to gather more recent data on these genetic markers in the underrepresented provinces.

To address the disparities in research distribution and the underrepresentation of northern and central provinces in Mozambique, future studies should prioritize comprehensive investigations into molecular markers of antimalarial resistance in regions with high malaria incidence, such as Zambezia, Nampula, and Cabo Delgado. These provinces are not only heavily affected by the disease, but also exhibit unique climatic and socio-economic conditions that may influence resistance patterns.

Expanding research into these areas will provide critical insights into the regional dynamics of resistance, enabling more targeted and effective malaria control strategies. Additionally, establishing collaborative research networks and strengthening local laboratory capacities in underrepresented provinces could ensure a more equitable distribution of scientific efforts. This approach will contribute to the development of a robust national framework for monitoring and combating antimalarial resistance, ultimately improving public health outcomes across the country.

## Figures and Tables

**Table 1:** Summary of the studies included in the review. SNP, single-nucleotide polymorphism; CNV, copy number variation.

Mutation	Gene	Province	Nº of Studies (2008–2024)	References	Year of Sample Collection *
**SNP**	***pfcrt***	**Maputo**	**6**	[10,50,51,52,53,54,55,56,57]	2015–2019 [51]
		**Gaza**	**2**		2015 [53]
		**Inhambane**	**1**		2018 [54]
		**Zambezia**			
		**Tete**			
		**Cabo Delgado**			
	***pfdhfr*, *pfdhps***	**Maputo**	**10**	[10,45,46,50,51,53,55,57,58,59,60,61,62]	2015–2019 [51]
		**Gaza**	**3**		2014–2015 [50]
		**Tete**	**1**		2015 [53]
		**Sofala**	**2**		
		**Cabo Delgado**	**1**		2015 [53]
	***pfmdr1***	**Maputo**	**5**	[10,50,51,52,53,54,55,57,63]	2015–2019 [51]
		**Gaza**	**3**		2014–2015 [50]
		**Inhambane**	**1**		2018 [54]
		**Zambezia**			
		**Tete**	**2**		
		**Sofala**	**1**		
		**Cabo Delgado**	**2**		
	***pfk13***	**Maputo**	**4**	[47,48,51,52,54]	2021 [47]
		**Gaza**	**1**		
		**Zambezia**			
		**Tete**			
		**Sofala**			
		**Manica**			
		**Cabo Delgado**			
		**Niassa**			
**CNV**	***pfpm2*/** ***pfpm3*/** ***pfmdr1***	**Maputo**	**2**	[52,53,64]	2021 [64]
		**Gaza**	**1**		2015 [53]
		**Tete**			
		**Sofala**			
		**Manica**			2021 [64]
		**Niassa**			

* indicates the year the samples were collected, providing the most recent data on the prevalence of the corresponding molecular marker.

## Data Availability

The authors confirm that the data supporting the findings of this study are available within the article and its Supplementary Materials.

## Supplementary Materials

The following supporting information can be downloaded at: https://www.mdpi.com/article/10.3390/ijms252413645/s1.

## Abbreviations

ART: artemisinin; LUM: lumefantrine; CQ: chloroquine; QN: quinine; PPQ: piperaquine; DHA: dihydroartemisinin; AQ: amodiaquine; MEF: mefloquine; PY: pyronaridine; SP: sulfadoxine-pyrimethamine; As: artesunate; AMT: artemether. SNP, single-nucleotide polymorphisms; CNV, copy number variation; ACT, artemisinin-based combination therapy.

## References

[1] Ippolito M.M., Moser K.A., Kabuya J.-B.B., Cunningham C., Juliano J.J. (2021). Antimalarial Drug Resistance and Implications for the WHO Global Technical Strategy. Curr. Epidemiol. Rep..

[2] Oladipo H.J., Tajudeen Y.A., Oladunjoye I.O., Yusuff S.I., Yusuf R.O., Oluwaseyi E.M., AbdulBasit M.O., Adebisi Y.A., El-Sherbini M.S. (2022). Increasing Challenges of Malaria Control in Sub-Saharan Africa: Priorities for Public Health Research and Policymakers. Ann. Med. Surg..

[3] Li J., Docile H.J., Fisher D., Pronyuk K., Zhao L. (2024). Current Status of Malaria Control and Elimination in Africa: Epidemiology, Diagnosis, Treatment, Progress and Challenges. J. Epidemiol. Glob. Health.

[4] (2023). WHO World Malaria Report. https://www.who.int/teams/global-malaria-programme/reports/world-malaria-report-2023.

[5] Hemingway J., Shretta R., Wells T.N.C., Bell D., Djimdé A.A., Achee N., Qi G. (2016). Tools and Strategies for Malaria Control and Elimination: What Do We Need to Achieve a Grand Convergence in Malaria?. PLoS Biol..

[6] Plowe C.V. (2022). Malaria Chemoprevention and Drug Resistance: A Review of the Literature and Policy Implications. Malar. J..

[7] Rasmussen C., Alonso P., Ringwald P. (2022). Current and Emerging Strategies to Combat Antimalarial Resistance. Expert. Rev. Anti Infect. Ther..

[8] Thu A.M., Phyo A.P., Landier J., Parker D.M., Nosten F.H. (2017). Combating Multidrug-resistant Plasmodium Falciparum Malaria. FEBS J..

[9] Wongsrichanalai C., Pickard A.L., Wernsdorfer W.H., Meshnick S.R. (2002). Epidemiology of Drug-Resistant Malaria. Lancet Infect. Dis..

[10] Raman J., Mauff K., Muianga P., Mussa A., Maharaj R., Barnes K.I. (2011). Five Years of Antimalarial Resistance Marker Surveillance in Gaza Province, Mozambique, Following Artemisinin-Based Combination Therapy Roll Out. PLoS ONE.

[11] Ecker A., Lehane A.M., Clain J., Fidock D.A. (2012). PfCRT and Its Role in Antimalarial Drug Resistance. Trends Parasitol..

[12] Mohammed A., Ndaro A., Kalinga A., Manjurano A., Mosha J.F., Mosha D.F., van Zwetselaar M., Koenderink J.B., Mosha F.W., Alifrangis M. (2013). Trends in Chloroquine Resistance Marker, Pfcrt-K76T Mutation Ten Years after Chloroquine Withdrawal in Tanzania. Malar. J..

[13] Foguim F.T., Bogreau H., Gendrot M., Mosnier J., Fonta I., Benoit N., Amalvict R., Madamet M., Wein S., Pradines B. (2020). Prevalence of Mutations in the Plasmodium Falciparum Chloroquine Resistance Transporter, PfCRT, and Association with Ex Vivo Susceptibility to Common Anti-Malarial Drugs against African Plasmodium Falciparum Isolates. Malar. J..

[14] Bygbjerg I.C., Alifrangis M., Tomás E.V.E., Charlwood D., Thomsen T.T., Madsen L.B., Hansson H.H. (2013). Rapid Selection of Plasmodium Falciparum Chloroquine Resistance Transporter Gene and Multidrug Resistance Gene-1 Haplotypes Associated with Past Chloroquine and Present Artemether-Lumefantrine Use in Inhambane District, Southern Mozambique. Am. J. Trop. Med. Hyg..

[15] Mehlotra R.K., Fujioka H., Roepe P.D., Janneh O., Ursos L.M.B., Jacobs-Lorena V., McNamara D.T., Bockarie M.J., Kazura J.W., Kyle D.E. (2001). Evolution of a Unique Plasmodium Falciparum Chloroquine-Resistance Phenotype in Association with Pfcrt Polymorphism in Papua New Guinea and South America. Proc. Natl. Acad. Sci. USA.

[16] Wootton J.C., Feng X., Ferdig M.T., Cooper R.A., Mu J., Baruch D.I., Magill A.J., Su X. (2002). Genetic Diversity and Chloroquine Selective Sweeps in Plasmodium Falciparum. Nature.

[17] Mishra N., Kaitholia K., Srivastava B., Shah N.K., Narayan J.P., Dev V., Phookan S., Anvikar A.R., Rana R., Bharti R.S. (2014). Declining efficacy of artesunate plus sulphadoxine-pyrimethamine in northeastern India. Malar. J..

[18] Jakubowski A., Stearns S.C., Kruk M.E., Angeles G., Thirumurthy H. (2017). The US President’s Malaria Initiative and under-5 child mortality in sub-Saharan Africa: A difference-in-differences analysis. PLoS Med..

[19] Esu E., Tacoli C., Gai P., Berens-Riha N., Pritsch M., Loescher T., Meremikwu M. (2018). Prevalence of the Pfdhfr and Pfdhps Mutations among Asymptomatic Pregnant Women in Southeast Nigeria. Parasitol. Res..

[20] Naidoo I., Roper C. (2013). Mapping ‘Partially Resistant’, ‘Fully Resistant’, and ‘Super Resistant’ Malaria. Trends Parasitol..

[21] Nhama A., Nhamússua L., Macete E., Bassat Q., Salvador C., Enosse S., Candrinho B., Carvalho E., Nhacolo A., Chidimatembue A. (2021). In Vivo Efficacy and Safety of Artemether–Lumefantrine and Amodiaquine–Artesunate for Uncomplicated Plasmodium Falciparum Malaria in Mozambique, 2018. Malar. J..

[22] Nsanzabana C. (2019). Resistance to Artemisinin Combination Therapies (ACTs): Do Not Forget the Partner Drug!. Trop. Med. Infect. Dis..

[23] Li Y., Li G., Li Y., Li Z., Zeng M. (2018). Artemisinin and Derivatives Pharmacodynamics, Toxicology, Pharmacokinetics, Mechanism of Action, Resistance, and Immune Regulation. Artemisinin-Based and Other Antimalarials.

[24] Dhorda M., Amaratunga C., Dondorp A.M. (2021). Artemisinin and Multidrug-Resistant Plasmodium Falciparum—A Threat for Malaria Control and Elimination. Curr. Opin. Infect. Dis..

[25] Wicht K.J., Mok S., Fidock D.A. (2020). Molecular Mechanisms of Drug Resistance in Plasmodium Falciparum Malaria. Annu. Rev. Microbiol..

[26] Phyo A.P., Ashley E.A., Anderson T.J.C., Bozdech Z., Carrara V.I., Sriprawat K., Nair S., White M.M., Dziekan J., Ling C. (2016). Declining Efficacy of Artemisinin Combination Therapy Against *P. falciparum* Malaria on the Thai–Myanmar Border (2003–2013): The Role of Parasite Genetic Factors. Clin. Infect. Dis..

[27] Price R.N., Uhlemann A.-C., Brockman A., McGready R., Ashley E., Phaipun L., Patel R., Laing K., Looareesuwan S., White N.J. (2004). Mefloquine Resistance in Plasmodium Falciparum and Increased Pfmdr1 Gene Copy Number. Lancet.

[28] Eyase F.L., Akala H.M., Ingasia L., Cheruiyot A., Omondi A., Okudo C., Juma D., Yeda R., Andagalu B., Wanja E. (2013). The Role of Pfmdr1 and Pfcrt in Changing Chloroquine, Amodiaquine, Mefloquine and Lumefantrine Susceptibility in Western-Kenya *P. falciparum* Samples during 2008–2011. PLoS ONE.

[29] Borges S., Cravo P., Creasey A., Fawcett R., Modrzynska K., Rodrigues L., Martinelli A., Hunt P. (2011). Genomewide Scan Reveals Amplification of Mdr1 as a Common Denominator of Resistance to Mefloquine, Lumefantrine, and Artemisinin in Plasmodium Chabaudi Malaria Parasites. Antimicrob. Agents Chemother..

[30] Preechapornkul P., Imwong M., Chotivanich K., Pongtavornpinyo W., Dondorp A.M., Day N.P.J., White N.J., Pukrittayakamee S. (2009). Plasmodium Falciparum Pfmdr1 Amplification, Mefloquine Resistance, and Parasite Fitness. Antimicrob. Agents Chemother..

[31] Sidhu A.B.S., Valderramos S.G., Fidock D.A. (2005). Pfmdr1 Mutations Contribute to Quinine Resistance and Enhance Mefloquine and Artemisinin Sensitivity in Plasmodium Falciparum. Mol. Microbiol..

[32] Póvoa M.M., Adagu I.S., Oliveira S.G., Machado R.L.D., Miles M.A., Warhurst D.C. (1998). Pfmdr1Asn1042AspandAsp1246TyrPolymorphisms, Thought to Be Associated with Chloroquine Resistance, Are Present in Chloroquine-Resistant and -Sensitive Brazilian Field Isolates OfPlasmodium Falciparum. Exp. Parasitol..

[33] Foote S.J., Kyle D.E., Martin R.K., Oduola A.M.J., Forsyth K., Kemp D.J., Cowman A.F. (1990). Several Alleles of the Multidrug-Resistance Gene Are Closely Linked to Chloroquine Resistance in Plasmodium Falciparum. Nature.

[34] Lekana-Douki J.B., Boutamba S.D.D., Zatra R., Edou S.E.Z., Ekomy H., Bisvigou U., Toure-Ndouo F.S. (2011). Increased Prevalence of the Plasmodium Falciparum Pfmdr1 86N Genotype among Field Isolates from Franceville, Gabon after Replacement of Chloroquine by Artemether–Lumefantrine and Artesunate–Mefloquine. Infect. Genet. Evol..

[35] Lopes D., Rungsihirunrat K., Nogueira F., Seugorn A., Gil J.P., do Rosário V.E., Cravo P. (2002). Molecular Characterisation of Drug-Resistant Plasmodium Falciparum from Thailand. Malar. J..

[36] Wurtz N., Fall B., Pascual A., Fall M., Baret E., Camara C., Nakoulima A., Diatta B., Fall K.B., Mbaye P.S. (2014). Role of Pfmdr1 in In Vitro Plasmodium Falciparum Susceptibility to Chloroquine, Quinine, Monodesethylamodiaquine, Mefloquine, Lumefantrine, and Dihydroartemisinin. Antimicrob. Agents Chemother..

[37] Dokomajilar C., Nsobya S.L., Greenhouse B., Rosenthal P.J., Dorsey G. (2006). Selection of Plasmodium Falciparum Pfmdr1 Alleles Following Therapy with Artemether-Lumefantrine in an Area of Uganda Where Malaria Is Highly Endemic. Antimicrob. Agents Chemother..

[38] Spring M.D., Lin J.T., Manning J.E., Vanachayangkul P., Somethy S., Bun R., Se Y., Chann S., Ittiverakul M., Sia-ngam P. (2015). Dihydroartemisinin-Piperaquine Failure Associated with a Triple Mutant Including Kelch13 C580Y in Cambodia: An Observational Cohort Study. Lancet Infect. Dis..

[39] Phuc B.Q., Rasmussen C., Duong T.T., Dong L.T., Loi M.A., Ménard D., Tarning J., Bustos D., Ringwald P., Galappaththy G.L. (2017). Treatment Failure of Dihydroartemisinin/ Piperaquine for Plasmodium Falciparum Malaria, Vietnam. Emerg. Infect. Dis..

[40] Kahunu G.M., Thomsen S.W., Thomsen L.W., Mavoko H.M., Mulopo P.M., Hocke E.F., Nkoli P.M., Baraka V., Minja D.T.R., Mousa A. (2024). Identification of the PfK13 Mutations R561H and P441L in the Democratic Republic of Congo. Int. J. Infect. Dis..

[41] Schmedes S.E., Patel D., Dhal S., Kelley J., Svigel S.S., Dimbu P.R., Adeothy A.-L., Kahunu G.M., Nkoli P.M., Beavogui A.H. (2021). Plasmodium Falciparum Kelch 13 Mutations, 9 Countries in Africa, 2014–2018. Emerg. Infect. Dis..

[42] Stokes B.H., Dhingra S.K., Rubiano K., Mok S., Straimer J., Gnädig N.F., Deni I., Schindler K.A., Bath J.R., Ward K.E. (2021). Plasmodium Falciparum K13 Mutations in Africa and Asia Impact Artemisinin Resistance and Parasite Fitness. Elife.

[43] Shamseer L., Moher D., Clarke M., Ghersi D., Liberati A., Petticrew M., Shekelle P., Stewart L.A. (2015). Preferred Reporting Items for Systematic Review and Meta-Analysis Protocols (PRISMA-P) 2015: Elaboration and Explanation. BMJ.

[44] Page M.J., McKenzie J.E., Bossuyt P.M., Boutron I., Hoffmann T.C., Mulrow C.D., Shamseer L., Tetzlaff J.M., Akl E.A., Brennan S.E. (2021). The PRISMA 2020 Statement: An Updated Guideline for Reporting Systematic Reviews. Syst. Rev..

[45] Allen E.N., Little F., Camba T., Cassam Y., Raman J., Boulle A., Barnes K.I. (2009). Efficacy of Sulphadoxine-Pyrimethamine with or without Artesunate for the Treatment of Uncomplicated Plasmodium Falciparum Malaria in Southern Mozambique: A Randomized Controlled Trial. Malar. J..

[46] Fernandes N., Figueiredo P., Do Rosário V.E., Cravo P. (2007). Analysis of Sulphadoxine/Pyrimethamine Resistance-Conferring Mutations of Plasmodium Falciparum from Mozambique Reveals the Absence of the Dihydrofolate Reductase 164L Mutant. Malar. J..

[47] da Silva C., Matias D., Dias B., Cancio B., Silva M., Viegas R., Chivale N., Luis S., Salvador C., Duarte D. (2023). Anti-Malarial Resistance in Mozambique: Absence of Plasmodium Falciparum Kelch 13 (K13) Propeller Domain Polymorphisms Associated with Resistance to Artemisinins. Malar. J..

[48] Escobar C., Pateira S., Lobo E., Lobo L., Teodosio R., Dias F., Fernandes N., Arez A.P., Varandas L., Nogueira F. (2015). Polymorphisms in Plasmodium Falciparum K13-Propeller in Angola and Mozambique after the Introduction of the ACTs. PLoS ONE.

[49] Kenangalem E., Poespoprodjo J.R., Douglas N.M., Burdam F.H., Gdeumana K., Chalfein F., Prayoga, Thio F., Devine A., Marfurt J. (2019). Malaria Morbidity and Mortality Following Introduction of a Universal Policy of Artemisinin-Based Treatment for Malaria in Papua, Indonesia: A Longitudinal Surveillance Study. PLoS Med..

[50] Galatas B., Nhamussua L., Candrinho B., Mabote L., Cisteró P., Gupta H., Rabinovich R., Menéndez C., MacEte E., Saute F. (2017). In-Vivo Efficacy of Chloroquine to Clear Asymptomatic Infections in Mozambican Adults: A Randomized, Placebo-Controlled Trial with Implications for Elimination Strategies. Sci. Rep..

[51] Raman J., Sharp B., Kleinschmidt I., Roper C., Streat E., Kelly V., Barnes K.I. (2008). Differential Effect of Regional Drug Pressure on Dihydrofolate Reductase and Dihydropteroate Synthetase Mutations in Southern Mozambique. Am. J. Trop. Med. Hyg..

[52] Gupta H., Galatas B., Chidimatembue A., Huijben S., Cisteró P., Matambisso G., Nhamussua L., Simone W., Bassat Q., Ménard D. (2020). Effect of Mass Dihydroartemisinin–Piperaquine Administration in Southern Mozambique on the Carriage of Molecular Markers of Antimalarial Resistance. PLoS One.

[53] Gupta H., Macete E., Bulo H., Salvador C., Warsame M., Carvalho E., Ménard D., Ringwald P., Bassat Q., Enosse S. (2018). Drug-Resistant Polymorphisms and Copy Numbers in Plasmodium Falciparum, Mozambique, 2015. Emerg. Infect. Dis..

[54] Chidimatembue A., Svigel S.S., Mayor A., Aíde P., Nhama A., Nhamussua L., Nhacolo A., Bassat Q., Salvador C., Enosse S. (2021). Molecular Surveillance for Polymorphisms Associated with Artemisinin-Based Combination Therapy Resistance in Plasmodium Falciparum Isolates Collected in Mozambique, 2018. Malar. J..

[55] Mayor A., Serra-Casas E., Sanz S., Aponte J.J., Macete E., Mandomando I., Puyol L., Berzosa P., Dobaño C., Aide P. (2008). Molecular Markers of Resistance to Sulfadoxine-Pyrimethamine during Intermittent Preventive Treatment for Malaria in Mozambican Infants. J. Infect. Dis..

[56] Casanova D., Baptista V., Costa M., Freitas B., Pereira M.d.N.I., Calçada C., Mota P., Kythrich O., Pereira M.H.J.S., Osório N.S. (2024). Artemisinin Resistance-Associated Gene Mutations in Plasmodium Falciparum: A Case Study of Severe Malaria from Mozambique. Travel Med. Infect. Dis..

[57] Huijben S., Macete E., Mombo-Ngoma G., Ramharter M., Kariuki S., Desai M., Shi Y.P., Mwangoka G., Massougbodji A., Cot M. (2020). Counter-Selection of Antimalarial Resistance Polymorphisms by Intermittent Preventive Treatment in Pregnancy. J. Infect. Dis..

[58] Raman J., Little F., Roper C., Kleinschmidt I., Cassam Y., Maharaj R., Barnes K.I. (2010). Five Years of Large-Scale Dhfr and Dhps Mutation Surveillance Following the Phased Implementation of Artesunate Plus Sulfadoxine-Pyrimethamine in Maputo Province, Southern Mozambique. Am. J. Trop. Med. Hyg..

[59] Enosse S., Magnussen P., Abacassamo F., Gómez-Olivé X., Rønn A.M., Thompson R., Alifrangis M. (2008). Rapid Increase of Plasmodium Falciparum Dhfr/Dhps Resistant Haplotypes, after the Adoption of Sulphadoxine-Pyrimethamine as First Line Treatment in 2002, in Southern Mozambique. Malar. J..

[60] Figueroa-Romero A., Bissombolo D., Meremikwu M., Ratsimbasoa A., Sacoor C., Arikpo I., Lemba E., Nhama A., Rakotosaona R., Llach M. (2023). Prevalence of Molecular Markers of Resistance to Sulfadoxine–Pyrimethamine before and after Community Delivery of Intermittent Preventive Treatment of Malaria in Pregnancy in Sub-Saharan Africa: A Multi-Country Evaluation. Lancet Glob. Health.

[61] Brokhattingen N., Matambisso G., da Silva C., Neubauer Vickers E., Pujol A., Mbeve H., Cisteró P., Maculuve S., Cuna B., Melembe C. (2024). Genomic Malaria Surveillance of Antenatal Care Users Detects Reduced Transmission Following Elimination Interventions in Mozambique. Nat. Commun..

[62] Matambisso G., Brokhattingen N., Maculuve S., Cístero P., Mbeve H., Escoda A., Bambo G., Cuna B., Melembe C., Ndimande N. (2024). Sustained Clinical Benefit of Malaria Chemoprevention with Sulfadoxine-Pyrimethamine (SP) in Pregnant Women in a Region with High SP Resistance Markers. J. Infect..

[63] Lobo E., de Sousa B., Rosa S., Figueiredo P., Lobo L., Pateira S., Fernandes N., Nogueira F. (2014). Prevalence of Pfmdr1 Alleles Associated with Artemether-Lumefantrine Tolerance/Resistance in Maputo before and after the Implementation of Artemisinin-Based Combination Therapy. Malar. J..

[64] Brown N., da Silva C., Webb C., Matias D., Dias B., Cancio B., Silva M., Viegas R., Salvador C., Chivale N. (2024). Antimalarial Resistance Risk in Mozambique Detected by a Novel Quadruplex Droplet Digital PCR Assay. Antimicrob. Agents Chemother..

[65] Mabunda S., Casimiro S., Quinto L., Alonso P. (2008). A country-wide malaria survey in Mozambique. I. Plasmodium falciparum infection in children in different epidemiological settings. Malar. J..

[66] Ocan M., Akena D., Nsobya S., Kamya M.R., Senono R., Kinengyere A.A., Obuku E.A. (2019). Persistence of Chloroquine Resistance Alleles in Malaria Endemic Countries: A Systematic Review of Burden and Risk Factors. Malar. J..

[67] Mang’era C.M., Mbai F.N., Omedo I.A., Mireji P.O., Omar S.A. (2012). Changes in Genotypes of Plasmodium Falciparum Human Malaria Parasite Following Withdrawal of Chloroquine in Tiwi, Kenya. Acta Trop..

[68] Mwanza S., Joshi S., Nambozi M., Chileshe J., Malunga P., Kabuya J.-B.B., Hachizovu S., Manyando C., Mulenga M., Laufer M. (2016). The Return of Chloroquine-Susceptible Plasmodium Falciparum Malaria in Zambia. Malar. J..

[69] Laufer M.K., Takala-Harrison S., Dzinjalamala F.K., Stine O.C., Taylor T.E., Plowe C.V. (2010). Return of Chloroquine-Susceptible Falciparum Malaria in Malawi Was a Reexpansion of Diverse Susceptible Parasites. J. Infect. Dis..

[70] Bwire G.M., Ngasala B., Mikomangwa W.P., Kilonzi M., Kamuhabwa A.A.R. (2020). Detection of Mutations Associated with Artemisinin Resistance at K13-Propeller Gene and a near Complete Return of Chloroquine Susceptible Falciparum Malaria in Southeast of Tanzania. Sci. Rep..

[71] Ross L.S., Dhingra S.K., Mok S., Yeo T., Wicht K.J., Kümpornsin K., Takala-Harrison S., Witkowski B., Fairhurst R.M., Ariey F. (2018). Emerging Southeast Asian PfCRT Mutations Confer Plasmodium Falciparum Resistance to the First-Line Antimalarial Piperaquine. Nat. Commun..

[72] Orlando P. (2019). Evaluation of Intermittent Preventive Treatment during Pregnancy (IPTp) in Chókwè District, Southern Mozambique: Coverage and Effect on Pregnancy and Parasitological Outcomes.

[73] Arnaldo P., Rovira-Vallbona E., Langa J.S., Salvador C., Guetens P., Chiheb D., Xavier B., Kestens L., Enosse S.M., Rosanas-Urgell A. (2018). Uptake of Intermittent Preventive Treatment and Pregnancy Outcomes: Health Facilities and Community Surveys in Chókwè District, Southern Mozambique. Malar. J..

[74] Kayode A.T., Ajogbasile F.V., Akano K., Uwanibe J.N., Oluniyi P.E., Eromon P.J., Folarin O.A., Sowunmi A., Wirth D.F., Happi C.T. (2021). Polymorphisms in Plasmodium Falciparum Dihydropteroate Synthetase and Dihydrofolate Reductase Genes in Nigerian Children with Uncomplicated Malaria Using High-Resolution Melting Technique. Sci. Rep..

[75] Duah N.O., Quashie N.B., Abuaku B.K., Sebeny P.J., Kronmann K.C., Koram K.A. (2012). Surveillance of Molecular Markers of Plasmodium Falciparum Resistance to Sulphadoxine-Pyrimethamine 5 Years after the Change of Malaria Treatment Policy in Ghana. Am. Soc. Trop. Med. Hyg..

[76] Duah N.O., Matrevi S.A., de Souza D.K., Binnah D.D., Tamakloe M.M., Opoku V.S., Onwona C.O., Narh C.A., Quashie N.B., Abuaku B. (2013). Increased Pfmdr1 Gene Copy Number and the Decline in Pfcrt and Pfmdr1 Resistance Alleles in Ghanaian Plasmodium Falciparum Isolates after the Change of Anti-Malarial Drug Treatment Policy. Malar. J..

[77] Njiro B.J., Mutagonda R.F., Chamani A.T., Mwakyandile T., Sabas D., Bwire G.M. (2022). Molecular Surveillance of Chloroquine-Resistant Plasmodium Falciparum in Sub-Saharan African Countries after Withdrawal of Chloroquine for Treatment of Uncomplicated Malaria: A Systematic Review. J. Infect. Public Health.

[78] Niba P.T.N., Nji A.M., Evehe M.-S., Ali I.M., Netongo P.M., Ngwafor R., Moyeh M.N., Ngum L.N., Ndum O.E., Acho F.A. (2021). Drug Resistance Markers within an Evolving Efficacy of Anti-Malarial Drugs in Cameroon: A Systematic Review and Meta-Analysis (1998–2020). Malar. J..

[79] Okell L.C., Reiter L.M., Ebbe L.S., Baraka V., Bisanzio D., Watson O.J., Bennett A., Verity R., Gething P., Roper C. (2018). Emerging Implications of Policies on Malaria Treatment: Genetic Changes in the *Pfmdr-1* Gene Affecting Susceptibility to Artemether–Lumefantrine and Artesunate–Amodiaquine in Africa. BMJ Glob. Health.

[80] Groger M., Veletzky L., Lalremruata A., Cattaneo C., Mischlinger J., Zoleko-Manego R., Endamne L., Klicpera A., Kim J., Nguyen T. (2018). Prospective Clinical Trial Assessing Species-Specific Efficacy of Artemether-Lumefantrine for the Treatment of Plasmodium Malariae, Plasmodium Ovale, and Mixed Plasmodium Malaria in Gabon. Antimicrob. Agents Chemother..

[81] Gaye A., Sy M., Ndiaye T., Siddle K.J., Park D.J., Deme A.B., Mbaye A., Dieye B., Ndiaye Y.D., Neafsey D.E. (2020). Amplicon Deep Sequencing of Kelch13 in Plasmodium Falciparum Isolates from Senegal. Malar. J..

[82] Jeang B., Zhong D., Lee M.-C., Atieli H., Yewhalaw D., Yan G. (2024). Molecular Surveillance of Kelch 13 Polymorphisms in Plasmodium Falciparum Isolates from Kenya and Ethiopia. Malar. J..

[83] da Silva C., Boene S., Datta D., Rovira-Vallbona E., Aranda-Díaz A., Cisteró P., Hathaway N., Tessema S., Chidimatembue A., Matambisso G. (2023). Targeted and Whole-Genome Sequencing Reveal a North-South Divide in *P. falciparum* Drug Resistance Markers and Genetic Structure in Mozambique. Commun. Biol..

[84] Leroy D., Macintyre F., Adoke Y., Ouoba S., Barry A., Mombo-Ngoma G., Ndong Ngomo J.M., Varo R., Dossou Y., Tshefu A.K. (2019). African Isolates Show a High Proportion of Multiple Copies of the Plasmodium Falciparum Plasmepsin-2 Gene, a Piperaquine Resistance Marker. Malar. J..

[85] Uwimana A., Umulisa N., Venkatesan M., Svigel S.S., Zhou Z., Munyaneza T., Habimana R.M., Rucogoza A., Moriarty L.F., Sandford R. (2021). Association of Plasmodium Falciparum Kelch13 R561H Genotypes with Delayed Parasite Clearance in Rwanda: An Open-Label, Single-Arm, Multicentre, Therapeutic Efficacy Study. Lancet Infect. Dis..

[86] Asua V., Conrad M.D., Aydemir O., Duvalsaint M., Legac J., Duarte E., Tumwebaze P., Chin D.M., Cooper R.A., Yeka A. (2021). Changing Prevalence of Potential Mediators of Aminoquinoline, Antifolate, and Artemisinin Resistance Across Uganda. J. Infect. Dis..

[87] Conrad M.D., Asua V., Garg S., Giesbrecht D., Niaré K., Smith S., Namuganga J.F., Katairo T., Legac J., Crudale R.M. (2023). Evolution of Partial Resistance to Artemisinins in Malaria Parasites in Uganda. N. Engl. J. Med..

[88] Greenwood B. (2023). Artemisinin-Resistant and HRP-Negative Malaria Parasites in Africa. N. Engl. J. Med..

[89] World Health Organization (2023). WHO Guidelines for Malaria—14 March 2023.

[90] Ndwiga L., Kimenyi K.M., Wamae K., Osoti V., Akinyi M., Omedo I., Ishengoma D.S., Duah-Quashie N., Andagalu B., Ghansah A. (2021). A Review of the Frequencies of Plasmodium Falciparum Kelch 13 Artemisinin Resistance Mutations in Africa. Int. J. Parasitol. Drugs Drug Resist..

[91] Von Wowern F., Makenga G., Wellmann Thomsen S., Wellmann Thomsen L., Filtenborg Hocke E., Baraka V., Opot B.H., Minja D.T.R., Lusingu J.P.A., Van-geertruyden J.-P. (2024). Lack of Selection of Antimalarial Drug Resistance Markers after Intermittent Preventive Treatment of Schoolchildren (IPTsc) against Malaria in Northeastern Tanzania. Int. J. Infect. Dis..

[92] Tajebe A., Aemero M., Francis K., Magoma G. (2015). Identification of Chloroquine Resistance Pfcrt-K76T and Determination of Pfmdr1-N86Y Copy Number by SYBR Green I QPCR. Asian Pac. J. Trop. Biomed..

[93] Ngalah B.S., Ingasia L.A., Cheruiyot A.C., Chebon L.J., Juma D.W., Muiruri P., Onyango I., Ogony J., Yeda R.A., Cheruiyot J. (2015). Analysis of Major Genome Loci Underlying Artemisinin Resistance and Pfmdr1 Copy Number in Pre- and Post-ACTs in Western Kenya. Sci. Rep..

[94] Tadele G., Jawara A., Oboh M., Oriero E., Dugassa S., Amambua-Ngwa A., Golassa L. (2023). Clinical Isolates of Uncomplicated Falciparum Malaria from High and Low Malaria Transmission Areas Show Distinct Pfcrt and Pfmdr1 Polymorphisms in Western Ethiopia. Malar. J..

[95] Agomo C.O., Mishra N., Olukosi Y.A., Gupta R., Kamlesh K., Aina O.O., Awolola S.T. (2021). Mutations in Pfcrt and Pfmdr1 Genes of Plasmodium Falciparum Isolates from Two Sites in Northcentral and Southwest Nigeria. Infect. Genet. Evol..

[96] Yobi D.M., Kayiba N.K., Mvumbi D.M., Boreux R., Kabututu P.Z., Situakibanza H.N.T., Umesumbu S.E., De Mol P., Speybroeck N., Mvumbi G.L. (2021). Assessment of Plasmodium Falciparum Anti-Malarial Drug Resistance Markers in Pfk13-Propeller, Pfcrt and Pfmdr1 Genes in Isolates from Treatment Failure Patients in Democratic Republic of Congo, 2018–2019. Malar. J..

[97] Liu Y., Liang X., Li J., Chen J., Huang H., Zheng Y., He J., Ehapo C.S., Eyi U.M., Yang P. (2022). Molecular Surveillance of Artemisinin-Based Combination Therapies Resistance in Plasmodium Falciparum Parasites from Bioko Island, Equatorial Guinea. Microbiol. Spectr..

[98] Ebong C., Sserwanga A., Namuganga J.F., Kapisi J., Mpimbaza A., Gonahasa S., Asua V., Gudoi S., Kigozi R., Tibenderana J. (2021). Efficacy and Safety of Artemether-Lumefantrine and Dihydroartemisinin-Piperaquine for the Treatment of Uncomplicated Plasmodium Falciparum Malaria and Prevalence of Molecular Markers Associated with Artemisinin and Partner Drug Resistance in Uganda. Malar. J..

[99] Koko V.S., Warsame M., Vonhm B., Jeuronlon M.K., Menard D., Ma L., Taweh F., Tehmeh L., Nyansaiye P., Pratt O.J. (2022). Artesunate–Amodiaquine and Artemether–Lumefantrine for the Treatment of Uncomplicated Falciparum Malaria in Liberia: In Vivo Efficacy and Frequency of Molecular Markers. Malar. J..

[100] Amaratunga C., Lim P., Suon S., Sreng S., Mao S., Sopha C., Sam B., Dek D., Try V., Amato R. (2016). Dihydroartemisinin–Piperaquine Resistance in Plasmodium Falciparum Malaria in Cambodia: A Multisite Prospective Cohort Study. Lancet Infect. Dis..

[101] Amato R., Lim P., Miotto O., Amaratunga C., Dek D., Pearson R.D., Almagro-Garcia J., Neal A.T., Sreng S., Suon S. (2017). Genetic Markers Associated with Dihydroartemisinin–Piperaquine Failure in Plasmodium Falciparum Malaria in Cambodia: A Genotype–Phenotype Association Study. Lancet Infect. Dis..

[102] Witkowski B., Duru V., Khim N., Ross L.S., Saintpierre B., Beghain J., Chy S., Kim S., Ke S., Kloeung N. (2017). A Surrogate Marker of Piperaquine-Resistant Plasmodium Falciparum Malaria: A Phenotype–Genotype Association Study. Lancet Infect. Dis..

[103] Arroz J.A.H. (2016). Increase in Cases of Malaria in Mozambique, 2014: Epidemic or New Endemic Pattern?. Rev. Saude Publica.

