# Supplementary material for: Mapping Antimalarial Drug Resistance in Mozambique: A Systematic Review of Plasmodium falciparum Genetic Markers Post-ACT Implementation
Source: Int J Mol Sci. 2024 Dec 20;25(24):13645. doi: 10.3390/ijms252413645 (PMC11728251; doi:10.3390/ijms252413645)
Supplement: Supplementary file 1 [file ijms-25-13645-s001.zip › ijms-3351524-supplementary.pdf]

Table S1. Prevalence of polymorphisms in the *pfcrt* gene. S, single SNP; IET, triple mutant (74I+75E and 76T) (CVIET, quintuple mutant (C72 + V73 + 74I+75E and 76T); CVMNK, quintuple mutant (C72 + V73 + M74+N75 and K76).

Autor	Sampling year	Province	Target group	Sample size	Gene	Polymorphism	Prevalence
Brokhattingen et al., 2024 [61]	2015-2019	Maputo	Children and Pregnant women	357	*pfcrt*	CVIET	1.10% (4/357)
Chidimatembue et al., 2021[54]	2018	Inhambane	Children	39	*pfcrt*	CVIET	0.00%
		Zambézia		38			0.00%
		Tete		12			0.00%
		Cabo Delgado		20			0.00%
Gupta et al, 2020 [52]	2017	Maputo	Pregnant women	104	*pfcrt*	66T	1.00% (1/104)
						74I	1.90% (2/104)
						75E	1.90% (2/104)
						76T	1.90% (2/104)
						120E	1.00% (1/104)
Huijben et al., 2020 [57]	2010-2013	Maputo	Pregnant women	151	*pfcrt*	CVMNK	53.60% (81/151)
						CVIET	46.40% (70/151)
Arnaldo, 2019 [73]	2014-2015	Maputo	Pregnant women	100	*pfcrt*	76T	12.70% (7/55)
Gupta et al., 2018 [53]	2018	Gaza	Children	351	*pfcrt*	CVIET	9.20% (8/87)
Galatas et al., 2017 [50]	1999	Maputo	Adults	108	*pfcrt*	76T	84.00% (91/108)
	2015			108	*pfcrt*	76T	0.90% (1/108)

Raman et al., 2011 [10]	2006	Gaza	Children	141	*pfcrt*	76T	96.50% (136/141)
	2007			38	*pfcrt*	76T	78.40% (37/38)
	2008			51	*pfcrt*	76T	88.20% (82/93)
	2009			230	*pfcrt*	76T	62.80% (32/51)
	2010			113	*pfcrt*	76T	19.20% (5/26)
Mayor et al., 2008 [55]	2007	Maputo	Children	77	*pfcrt*	76T	88.30 (68/77)

Table S2. Prevalence of polymorphisms in the *pfdhr* and *pfdhps* genes. S, single SNP; IRN, triple mutant (*pfdhfr* 51I + 59R + 108N or *pfdhps* 437G + 540E + 581G); IRNGE, quintuple mutant (*pfdhfr* 51I + 59R + 108N and *pfdhps* 437G + 540E; IRNGEG, sextuple mutant (*pfdhfr* 51I + 59R + 108N and *pfdhps* 437G + 540E + 581G or 613S/T.

Author	Sampled year	Province	Target group	Sample size	Gene	Polymorphism	Prevalence
Brokhattingen et al., 2024 [61]	2015-2019	Maputo	Children and Pregnant women	352	*pfdhfr*	51I	88.10(349/352)
				337		59R	98.80% (333/337)
				353		108N	99.20%(350/353)
				365	*pfdhps*	437G	96.20(351/365)
				355		540E	94.40% (335/355)
				286		IRN	98.60% (282/286)
				300		GE	95.00% (285/300)
				245	*pfdhr/pfdhps*	IRNGE	95.10% (233/245)
Matambisso et al., 2024 [62]	2016-2019	Maputo	Pregnant women	138	*pfdhfr*	51I	97.80% (135/138)
						59R	98.80 (136/138)
						108N	98.80 (136/138)
					pfdhps	437G	94.90 (131/138)
						540E	94.90 (131/138)
					pfdhr/Pfdhps	IRNGE	94.20(130/138)
Figueroa-Romero et al., 2023 [60]	2015	Sofala	Children	301	*pfdhfr*	IRN	74.00% (222/301)
					pfdhps	GE	83.00% (251/301)
					pfdhr/pfdhps	IRNG	61.00% (183/301)
						IRNGE	0.00% (0/301)
	2010 -2013	Maputo		153	*pfdhr/pfdhps*	IRN	5.90% (9/153)

Huijben et al., 2020 [57]			Pregnant women			IRNG	0.70% (1/153)
						IRNGE	89.50% (137/153)
Arnaldo, 2019 [73]	2014 -2015	Gaza	Pregnant women	100	*Pfdhfr*	51I	89.00% (79/100)
						59R	86.00% (79/100)
						108N	90.00% (79/100)
						164L	2.00% (79/100)
					Pfdhps	437G	74.00% (79/100)
						540E	79.00% (79/100)
						581G	14.00% (14/100)
						IRN	90.00% (90/100)
				14	*pfdhr/pfdhps*	GEG	78.50% (11/14)
				100	*pfdhr/pfdhps*	GE	72.00% (72/100)
						IRNGEG	8.00% (8/100)
						IRNGE	55.00% (55/100)
Gupta et al., 2018 [53]	2015	Maputo	Children	153	*pfdhr/pfdhps*	IRN	5.90% (9/153)
						IRNG	0.70%(1/153)
						IRNGE	89.50% (137/153)
		Gaza		87	*pfdhps*	436F	2.4 0% (2/87)
						437G	90.80% (79/87)
						540E	88.50% (77/87)
		Tete		89	*pfdhps*	437G	87.50 % (77/88)
						540E	86.40 % (76/88)
					pfdhr/pfdhps	GE	86.40 % (76/88)
						IRNGE	0.00% (0/89)
		Sofala		88	*pfdhps*	437G	92.10 % (82/89)
						K540E	93.20% (83/89)

						GE	89.90% (80/89)
					pfdhr/pfdhps	GE	88.50% (77/87)
		Cabo Delgado		87	*pfdhps*	436F	34.50% (30/87)
						437G	58.60 % (51/87)
						540E	57.50 % (50/87)
					pfdhr/pfdhps	GE	0.0% (0/87)
						IRNGE	54.10 % (47/87)
Raman et al., 2011 [10]	2006	Gaza	Children	309	*pfdhr/pfdhps*	IRNGE	37.50% (116/309)
	2007			52		IRNGE	36.50% (19/52)
	2008			101		IRNGE	83.20% (84/101)
	2009			93		IRNGE	82.80% (77/93)
	2010			44		IRNGE	68.20% (30/44)
Raman et al., 2010 [58]	2004 and 2008	Maputo	Children	2012	*pfdhps*	S436S	0.00% (6/2012)
					pfdhr/pfdhps	GE	75.00% (1509/2,012)
					Pfdhr/Pfdhps	IRNGE	75.00% (1509/2,012)
Allen et al., 2009 [45]	2003 to 2005	Maputo	Children and adults	195	*pfdhr/pfdhps*	IRNGE	23.1% (45/195)
Enosse et al., 2008 [59]	2002-2003	Maputo	Children	133	*pfdhfr*	51I	36.60 (49/133)
				126		59R	52.4 (66/126)
				127		108N	50.40 (64/127)
				126	*pfdhps*	540E	7.90% (10/126)
Mayor et al., 2008 [55]	2007	Maputo	Children	77	*Pfdhfr*	51I	71.00% (55/77)
						59R	90.00%(69/77)
						108N	94.00%(72/77)
					pfdhps	437G	42.00% (32/77)

						540E	44.00% (34/77)
					pfdhr/pfdhps	IRNGE	26.00% (20/77)
Raman et al., 2008 [51]	1999, 2001, 2003, and 2004	Maputo	Children and adolescents	1114	*pfdhps*	436F	0.01% (11/1114)
						581G	0.00% (1/1114)
					pfdhfr	IRN	96.00% (1069/1114)
Fernandes et al., 2007 [46]	2004	Maputo	Children and adolescents	92	*pfdhfr*	51I	88.00% (81/92)
						59R	91.00% (84/92)
						108N	95.00% (87/92)
					pfdhps	437G	53.00% (49/92)
						540E	40.00% (37/92)
					pfdhr/pfdhps	IRNG	66.00% (61/92)
						IRNGE	47.00% (43/92)

Table S3. Prevalence of polymorphisms in the *pfmdr1* gene S, single SNP; NFD, triple mutant (N86+184F and 1246D)

Author	Sampled year	Province	Target group	Sample size	Gene	Polymorphism	Prevalence
Brokhattingen et al., 2024 [61]	2015-2019	Maputo	Children and Pregnant women	335	*pfmdr1*	N86	98.80% (331/335)
						86Y	1.20% (4/335)
				333		Y184	75.40% (251/333)
				377		D1246	99.70 (376/377)
						1246Y	0.30% (1/377)
Chidimatembue et al,2021 [54]	2018	Inhambane	Children	39	*pfmdr1*	Y184	25.60% (10/39)
						184F	53.80% (21/39)
						Y/F	20.50% (8/39)
						NFD	74.40 % (29/39)
		Zambezia		38		Y184	50.00% (19/38)
						184F	31.60% (12/38)
						Y/F	18.40% (7/38)
						NFD	50.00% (19/38)
		Tete		12		Y184	45.00% (9/20)
						184F	25.00% (5/20)
						Y/F	30.00% (6/20)
						NFD	11.00% (11/20)
		Cabo Delgado		20		Y184	33.30% (4/12)
						184F	41.70% (5/12)
						Y/F	25.00% (3/12)
						NFD	66.70% (8/12)
Gupta et al,2020 [52]	2017	Maputo	Pregnant women	105	*pfmdr1*	160K	3.80% (4/105)
						86Y	1.00% (1/105)

						G102	2.90 % (3/105)
						G182	5.70 (6/105)
						184F	50.50 % (53/105)
						1034C	2.90% (3/105)
						1042D	1.90% (2/105)
						T1069	1.00% (1/105)
						D1179	1.90% (2/105)
						Q1195	1.90% (2/105)
						1246Y	1.90% (2/105)
						1214L	1.90% (2/105)
						1197N	1.90% (2/105)
Huijben et al., 2020 [57]	2010-2013	Maputo	Pregnant women	136	*pfmdr1*	86Y	16.20% (22/136)
						184F	41.40 (60/136)
						Y/F	4.40% (6/136)
Arnaldo, 2019 [73]	2019	Gaza	Pregnant women	70	*pfmdr1*	N86	95.70% (67/70)
						86Y	4.30% (3/70)
						184F	40.00% (28/70)
						D1246	95.70% (67/70)
						1246Y	4.30% (3/70)
Gupta et al., 2018 [53]	2015	Gaza	Children	87	*pfmdr1*	N86	93.10% (81/87)
						86Y	6.90 % (6/87)
						G102	2.30 % (2/87)
						G182	1.10% (1/87)
						184F	44.80% (49/87)
						D1061	1.10 % (1/87)
						T1069	5.70 % (5/87)

						D1179	10.30% (9/87)
						1192A	1.10 % (1/87)
		Tete		89		G102	2.20% (2/89)
						G182	3.40% (3/89)
						184F	47.20 % (42/89)
						L1030	1.10 % (1/89)
						T1069	4.50 % (4/89)
						D1127	2.20 % (2/89)
						D1179	2.20% (2/89)
						N1189	1.10% (1/89)
		Sofala		88		N88	95.50% (84/88)
						86Y	4.50 % (4/88)
						G102	1.10% (1/88)
						G182	1.10% (1/88)
						184F	43.20% (38/88)
						T1069	9.20% (8/88)
						S1137S	2.30% (2/88)
						N1189	1.10% (1/88)
						1197N	1.10% (1/88)
		Cabo Delgado		87		86Y	1.10 % (1/87)
						G102	1.10% (1/87)
						G182	4.60% (4/87)
						184F	51.70% (45/87)
						T1069	11.50% (10/87)
						D1127	1.10% (1/87)
						S1137	2.30% (2/87)

						L1174	1.10% (1/87)
						1194S	1.10% (1/87)
						1197N	1.10% (1/87)
Lobo et al., 2014 [63]	2003-2005	Maputo	Adults	133	*pfmfr1*	184Y	58.70 (78/133)
				62		184F	19.60 (12/62)
	2010-2012			340		N86Y	14.10% (48/340)
				348		184F	22.90% (80/348)
				337		1246Y	2.40% (8/337)
Raman et al., 2011 [10]	2006	Gaza	Children and adolescents	307	*pfmdr1*	86Y	79.50% (244/307)
	2008			51		86Y	78.40% (40/51)
	2009			99		86Y	82.80% (82/99)
	2010			43		86Y	48.80% (21/43)
Mayor et al., 2008 [55]	2007	Maputo	Children	77	*pfmfr1*	86Y	57.00% (44/77)

Table S4. Prevalence of polymorphisms in the *pfk13* gene S, single SNP;

Author	Sampled year	Province	Target group	Sample size	Gene	Polymorphism	Prevalence
Brokhattingen et al., 2024 [61]	2015-2019	Maputo	Children and Pregnant women	343	*pfk13*	446I, 458Y, 476I, 493H	0% (0/343)
				341	*pfk13*	539T, 543T, 553L, 561H, 580Y	0.00% (0/341)
da Silva et al,2023 [83]	2015	Maputo	Children and adults	1034	*pfk13*	372E	0.00 (1/1034)
						598S	0.00 (1/1034
						599S	0.00 (1/1034
						641N	0.00 (1/1034
						659G	0.00 (1/1034
	2021			100	*pfk13*	663I	0.01% (1/100)
						661H	0.01% (1/100)
						543S	0.01% (1/100)
						455Q	0.01% (1/100)
	2018	Maputo		1034		384R	0.00 (1/1034
						386L	0.00 (1/1034
						442L	0.00 (1/1034
						483L	0.00 (1/1034
						494I	0.00 (1/1034
						520F	0.00 (1/1034
						537D	0.00 (1/1034
						605G	0.00 (1/1034
						607E	0.00 (1/1034

						631F	0.00 (1/1034
	2015	Gaza		1034		436S	0.00 (1/1034
						480R	0.00 (1/1034
						483S	0.00 (1/1034
						553T	0.00 (1/1034
	2018	Tete		100		578S	4.00% (4/100)
	2015	Sofala		440		386I	0.00 (1/440)
						546H	0.00 (1/440)
						656I	0.00 (1/440)
	2021	Manica		100		449R	0.01% (1/98)
						464Y	0.01% (1/100)
						470R	0.02% (2/98)
						509D	0.01% (1/98)
						654H	0.01% (1/98)
						S459	0.03% (3/98)
						C469	0.03% (3/98)
						G548	0.01% (1/98)
						L598	0.01% (1/98)
						G549	0.01% (1/98)
	2021	Niassa		98		454E	0.01% (1/100)
						464H	0.01% (1/100)
						494L	0.01% (1/100)
						532W	0.01% (1/100)
						578S	0.01% (1/100)
						578P	0.01% (1/100)
						581A	0.01% (1/100)

	2015	Cabo Delgado		100	*pfk13*	404K	0.01% (1/100)
						454I	0.01% (1/100)
						485G	0.01% (1/100)
						544R	0.01% (1/100)
						661R	0.01% (1/100)
						662S	0.01% (1/100)
	2018					599D	0.01% (1/100)
Chidimatembue et al,2021[54]	2017	Zambezia	Children	109	*pfk13*	G548	2.80% (3/109)
						C469	0.90 % (1/109)
Gupta et al,2020 [52]	2015 e 2017	Maputo	Pregnant women	112	*pfk13*	S477	0.90% (1/112)
						G548	1.80% (2/112)
						G690	1.80% (2/112)
Escobar et al., 2015 [48]	2010-2012	Maputo	Adults	200	*pfk13*	494I	4.00% (8/200)

Table S5. Prevalence of copy number variations (CNV) *pfmdr1*, *pfpm2* and *pfpm3*.

Author	Sampled year	Province	Target group	Sample size	Gene	Prevalence (CNV>1) Prevalence
Brown et al., 2024 [64]	2023	Maputo	Children and adults	79	*pfmdr1*	5.70 (5/79)
					pfpm2	3.40% (3/79)
					pfpm3	2.30% (2/79)
		Manica		87	*pfmdr1*	1.10% (1/87)
					pfpm2	1.10% (1/87)
					pfpm3	2.30% (1/87)
		Niassa		63	*pfmdr1*	4.80% 3/63)
					pfpm2	1.60% (1/63)
					pfpm3	1.60% (1/63)
Gupta et al,2020 [52]	2017	Maputo	Pregnant women	59	*pfmdr1*	5.10% (4/59)
					pfpm2	3.40% (2/59)
Gupta et al., 2018 [53]	2015	Gaza	Children	87	*pfmdr1*	2.30% (2/87)
					pfpm2	2.30% (2/87)
		Tete		89	*pfmdr1*	1.10% (1/89)
					pfpm2	1.10% (1/89)
		Sofala		88	*pfmdr1*	2.30% (2/88)
					pfpm2	1.10% (1/88)
